# Kyste thymique: étiologie rare d’un kyste cervical de l’adulte

**DOI:** 10.11604/pamj.2017.28.136.12493

**Published:** 2017-10-11

**Authors:** Souha Kallel, Malek Mnejja

**Affiliations:** 1 Service ORL et Chirurgie Cervico-Faciale, CHU Habib Bourguiba, 3029 Sfax, Tunisie

**Keywords:** Thymus, kyste, cou, médiastin, Thymus, cyst, neck, mediastinum

## Image en médecine

Les kystes thymiques sont des tumeurs congénitales rares, présentant 1% des masses cervicales kystiques de l'enfant. Ces kystes sont extrêmement rares chez l'adulte. Nous rapportons le cas d'une femme âgée de 50 ans, sans antécédents particuliers, qui a consulté pour une tuméfaction basi-cervicale antérieure évoluant progressivement depuis 2 ans avec une dyspnée d'effort. A l'examen, elle présentait une tuméfaction basi-cervicale antérieure faisant 4 * 4cm de diamètre, molle, non mobile à la déglutition. L'échographie cervicale a montré une formation kystique ovalaire sous cutanée du creux sus sternal plongeant au niveau du médiastin de 4 * 2,5 * 3cm de dimensions. Le complément par une TDM cervico-thoracique a montré une masse hypodense cervico-médiastinale médiane venant au contact des troncs supra aortiques, refoulant la thyroïde en haut et se développant en bas vers la loge thymique, mesurant 46 * 64 * 58mm. L'exploration chirurgicale par cervicotomie a trouvé une masse kystique tendue bien encapsulée de la loge thyroïdienne étendue au médiastin antérieur, à contenu jaune citrin. La masse était extirpée complètement par voie cervicale. L'étude histologique de la pièce opératoire était en faveur d'un kyste thymique. Le recul est de 1 an avec une bonne évolution sans récidive. Les kystes thymiques, bien que rares, doivent être évoqués devant toute masse kystique cervicale ou cervico-médiastinale, même de l'adulte. La TDM, l'IRM et la cytoponction préopératoires sont utiles, mais la confirmation diagnostique nécessite l'identification du tissu thymique contenant les corpuscules de Hassall à l'examen anatomopathologique. Le traitement est chirurgical avec une exérèse complète de la paroi kystique afin d'éviter les récidives.

**Figure 1:**
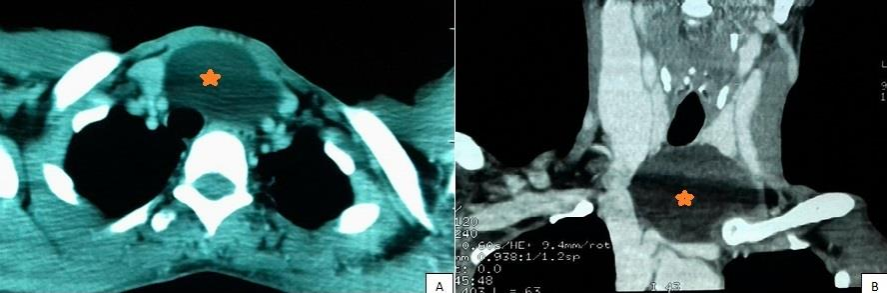
(A) scanner cervico-thoracique en coupe axiale; (B) coronale: masse hypodense cervico-médiastinale bien limitée, médiane venant au contact des troncs supra aortiques, refoulant la thyroïde en haut et se développant en bas vers la loge thymique, mesurant 46 * 64 * 58mm

